# Development of the International Classification of Diseases Ontology (ICDO) and its application for COVID-19 diagnostic data analysis

**DOI:** 10.1186/s12859-021-04402-2

**Published:** 2021-10-18

**Authors:** Ling Wan, Justin Song, Virginia He, Jennifer Roman, Grace Whah, Suyuan Peng, Luxia Zhang, Yongqun He

**Affiliations:** 1 University of Michigan Medical School, Ann Arbor, MI 48109 USA; 2 OntoWise, Nanjing, Jiangsu China; 3 Cranbrook Kingswood Upper School, Bloomfield Hills, MI 48304 USA; 4 Huron High School, Ann Arbor, MI 48105 USA; 5 College of Literacy, Science, and Arts, University of Michigan, Ann Arbor, MI 48109 USA; 6 College of Engineering, University of Michigan, Ann Arbor, MI 48109 USA; 7 School of Public Health, Peking University, Beijing, China; 8 National Institute of Health Data Science, Peking University, Beijing, China; 9 Advanced Institute of Information Technology, Peking University, Hangzhou, China; 10 Renal Division, Department of Medicine, Peking University First Hospital, Peking University Institute of Nephrology, Beijing, China

**Keywords:** ICD, Ontology, ICDO, Disease standardization, COVID-19, Bioinformatics

## Abstract

**Background:**

The 10th and 9th revisions of the International Statistical Classification of Diseases and Related Health Problems (ICD10 and ICD9) have been adopted worldwide as a well-recognized norm to share codes for diseases, signs and symptoms, abnormal findings, etc. The international Consortium for Clinical Characterization of COVID-19 by EHR (4CE) website stores diagnosis COVID-19 disease data using ICD10 and ICD9 codes. However, the ICD systems are difficult to decode due to their many shortcomings, which can be addressed using ontology.

**Methods:**

An ICD ontology (ICDO) was developed to logically and scientifically represent ICD terms and their relations among different ICD terms. ICDO is also aligned with the Basic Formal Ontology (BFO) and reuses terms from existing ontologies. As a use case, the ICD10 and ICD9 diagnosis data from the 4CE website were extracted, mapped to ICDO, and analyzed using ICDO.

**Results:**

We have developed the ICDO to ontologize the ICD terms and relations. Different from existing disease ontologies, all ICD diseases in ICDO are defined as disease processes to describe their occurrence with other properties. The ICDO decomposes each disease term into different components, including anatomic entities, process profiles, etiological causes, output phenotype, etc. Over 900 ICD terms have been represented in ICDO. Many ICDO terms are presented in both English and Chinese. The ICD10/ICD9-based diagnosis data of over 27,000 COVID-19 patients from 5 countries were extracted from the 4CE. A total of 917 COVID-19-related disease codes, each of which were associated with 1 or more cases in the 4CE dataset, were mapped to ICDO and further analyzed using the ICDO logical annotations. Our study showed that COVID-19 targeted multiple systems and organs such as the lung, heart, and kidney. Different acute and chronic kidney phenotypes were identified. Some kidney diseases appeared to result from other diseases, such as diabetes. Some of the findings could only be easily found using ICDO instead of ICD9/10.

**Conclusions:**

ICDO was developed to ontologize ICD10/10 codes and applied to study COVID-19 patient diagnosis data. Our findings showed that ICDO provides a semantic platform for more accurate detection of disease profiles.

## Background

The International Classification of Diseases (ICD), maintained by the World Health Organization (WHO), is the international diagnostic classification standard for reporting diseases and health conditions and for different clinical and research purposes. ICD defines diseases, disorders, injuries, and other related health conditions in the biomedical and clinical domains in a comprehensive and hierarchical fashion. The ICD has been continuously revised and published in a series of editions to reflect advances in health and medical science over time [1, 2], and is the foundation for sharing scrupulous statistics and identifying faultless health trends in the global medical and health community. The standardized data is crucially important to avoid purported pathogenic information that might cause misleading curative methods or disease prevention, especially while dealing with a new and unknown virus, COVID-19.

Many countries have adopted the ICD standard and developed their own modified versions, for instance, the USA version of ICD-10-CM [3] and the German version of *ICD*-10-GM [4]. In China, there are different formats, including National Standard V.1.1, GB/T14396-2016, and National Clinical Version 1.1. The availability of so many versions makes it difficult to standardize health records in China. This study focuses on the GB/T14396-2016, which is the ICD10 Chinese version authorized by a Chinese government agency. Recently WHO released the ICD11, which will officially come into effect on 1 January 2022 by WHO, and China was reported to adopt the ICD11 version as soon as it is ready. The ICD is used as the controlled terminology of diseases in the medical information platform in most healthcare administrations. There are many application systems that exist in hospitals, such as health information systems (HIS) [5], laboratory information system (LIS) [6], a picture archiving and communication system (PACS), and the electronic medical records (EMR). All these data can be integrated by the ICD framework. On the other hand, both ICD codes and diagnosis-related groups (DRGs) are major methods for medical insurance control, and the implementation of the DRGs is dependent on the correctness of ICD [7]. Due to its important role in many medical and clinical fields, a massive amount of mapping effort is required to ensure interoperability among different ICD versions.

The semantic mapping among databases generated under two different coding systems (e.g., ICD10 and ICD11) is very difficult and generally requires manual intervention. The National Institutes of Health (NIH) refers to such difficulty to the phenomenon of ‘data wrangling’ encompassing activities that make data more usable by changing their forms but not their meanings [8]. Although great efforts have been made in this area, the obstacle still exists. The ICD terminology is composed of a code/value pair. Each ICD standard code corresponds to a unique disease name as a value. However, in reality, there are often multiple synonyms expressed for one disease in the natural language. For example, the ICD11 code AA0Z has the value of “Infectious diseases of the external ear, unspecified”; the GB/T14396-2016 code H60.001 has the value of 外耳疖 (external ear furuncle); the ICD10 code H60.5 has the value of acute otitis externa, noninfective. Due to the existence of polysemy in natural language (especially in Chinese), the code-value mapping often encounters ambiguity after using the Extraction-Transfer-Load (ETL) tool for data integration and results in improper matching. Particularly in China, these problems are mainly due to the different local ICD versions with private extensions to certain ICD terms. These modifications are made according to the internal clinical needs coming from different medical units. The large discrepancy among different versions could cause many problems, such as the appearance of a large amount of data with different values but the same code or the same value with different codes. This also affects the accuracy of ICD-based DRG grouping, the accuracy of Medicare payments as well as the accuracy of the statistics of death causes.

ICD10 has been used for COVID-19 disease coding. Some COVID-19-specific codes have been recently added to ICD10. The new and old ICD10 has been used for clinical COVID-19 case reports. For example, the Consortium for Clinical Characterization of COVID-19 by EHR (4CE, https://covidclinical.net/) is an international consortium for the study of the COVID-19 pandemic by utilizing electronic health record (EHR) data [9]. As of 5 July 2020, 4CE collected clinical data of 27,584 COVID-19 cases from 95 hospitals in five countries of the USA, France, UK, Italy, and Singapore, which represent three continents of North America, Europe, and Asia, respectively. The disease symptoms from these patients were coded using ICD10 and ICD9 codes. The usage of the ICD codes supported the data standardization and sharing. However, how to transform the ICD data to a meaningful representation of COVID-19 appears to be a significant challenge.

In addition to the ICD, there are many disease description models being developed and used. Hadzic et al. classify a disease into four dimensions: (1) generic disease types; (2) phenotypes that are mainly based on observations to describe the various symptoms of the disease; (3) etiology that is a strictly scientific basis of pathogenic factors, mainly including two categories—genetic factors and environmental factors; (4) treatment that is a possible effective measure against a particular disease [10]. These four dimensions together can describe the overall knowledge of a disease field. On the basis of the axis, the general disease description model of Hadzic was improved, and two basic characteristics of complications and detection methods were added, and the symptoms, signs, staging, sex, age, acute and chronic, and onset time were classified as clinical manifestations [11].

Ontology is likely the best approach to solve the issue of semantic mapping among different databases and terminology systems. A formal biomedical ontology is a set of computer and human-interpretable terms that represent entities and relations in a biomedical domain. Ontologies have emerged to be critical to biomedical and clinical data standardization, management, integration, and analysis. Two different databases or terminologies may be formed based on different organizational principles and are unlikely or difficult to form an agreement about what each piece of information refers to and how they can be aligned. The inability to achieve interoperability can severely compromise the goals of information integration and aggregation. Such an issue is difficult to solve internally or among the two databases [8]. The usage of community-based and consensus-based ontologies supports information integration and solve the issue of term mapping.

Many disease-related ontologies exist, including Human Disease Ontology (DOID) [12, 13], Monarch Disease Ontology (MONDO) [14], and the Ontology of General Medical Science (OGMS) [15]. In DOID and MONDO, diseases are treated as disposition, which is a realizable entity that bears in some material entity and can be realized in a life process [8]. However, in the setting of ICD usage, diseases have already occurred and are not dispositions but rather processes. OGMS includes two high-level terms: disease and ‘disease course’, where the disease is asserted as a disposition and ‘disease course’ as a process.

To find a semantic mapping method between different ICD versions, here we report the development of an ICD ontology (ICDO) to address the issues of database interoperability and data integration, as listed above. Given that ICD is mainly applicable to statistical analysis and disease grouping for healthcare insurance, we present in this paper, our disease design pattern that combines the advantages of the above disease description models. Our disease design pattern in ICDO is based on the understanding that the disease in ICD is a human pathological process that realizes disease disposition. Such a process is composed of a group of entities, which has reversible decomposition. These entities are ‘anatomical structure’, ‘pathological anatomical entity’, ‘etiology’, ‘disease profile’, and ‘phenotype’. Therefore, all the ICD terms are defined as subclasses of the ICDO ‘disease process’ class, which is then defined as a subclass of the imported OGMS term ‘pathological bodily process’ [15].

In this manuscript, we detail our ICDO developmental strategy and provide a comprehensive use case to illustrate the usage of the ICDO. Note that the initial development of the ICDO was presented at the 10th International Conference on Biomedical Ontology (ICBO-2019) [16]. The further development of the ICDO and its application for COVID-19 data analysis use case study were represented in the 19th International Conference on Bioinformatics (InCoB 2020) [17].

## Methods

### General ICDO development strategy

Our ICDO development closely followed the WHO ICD 10/11 classification and principles. The ICDO development used the eXtensible Ontology Development (XOD) strategy [18], which emphasizes the reuse and alignment of ontology terms and semantic relations, ontology design patterns, and community effort. Specifically, we aligned the ICDO terms with Basic Formal Ontology (BFO) and BFO-compatible ontologies [8]. Ontofox [19] was used to extract terms from existing ontologies that were then imported and reused in ICDO.

We focused our first stage of ICDO development on two use cases, one is the representation of the specific area of external ear diseases, and the other focusing on the representation of approximately 400 ICD10/ICD9 codes that were used for COVID-19 diagnosis by the 4CE organization [9]. The first stage ICDO prototype covered all diseases related to external ear part in ICD11 under the class “Disease of the ear and mastoid process” (AA00 to AA6Z), ICD10 under the “external ear diseases”, and GB/T 14396-2016 (a commonly used ICD10 system in China. The second stage of ICDO development included over 400 ICD10/9 codes used to represent the COVID-19 associated diseases represented in the 4CE project. Only the second use case is introduced in this manuscript.

The Protégé OWL editor (http://protege.stanford.edu) was used to visualize ICDO, add new ICDO terms, edit imported terms, and merge imported ontologies. ICDO-specific terms were generated using new ICDO identifiers with the prefix “ICDO_” followed by 7-digit auto-incremented numbers. The Hermit reasoner was used for consistency checking and reasoning (http://hermit-reasoner.com/). In addition to the usage of the reasoner, ICDO was also evaluated using many other methods, including its comparison with the ICD-10 and ICD-9, the usage of references for ICD terms, its usages in different applications, and the feedback from other ICDO users [20].

### ICDO format, source code, and deposition

ICDO is expressed using the W3C standard Web Ontology Language (OWL2) (http://www.w3.org/TR/owl-guide/). The current ICDO source code is openly available at GitHub: http://github.com/icdo/ICDO.

The ICDO ontology is deposited in the NCBO BioPortal website: https://bioportal.bioontology.org/ontologies/ICDO, as well as the ontology repository website Ontobee [21]: http://www.ontobee.org/ontology/ICDO.

### Applications of ICDO for COVID-19 disease classifications and analyses

**Fig. 1:**
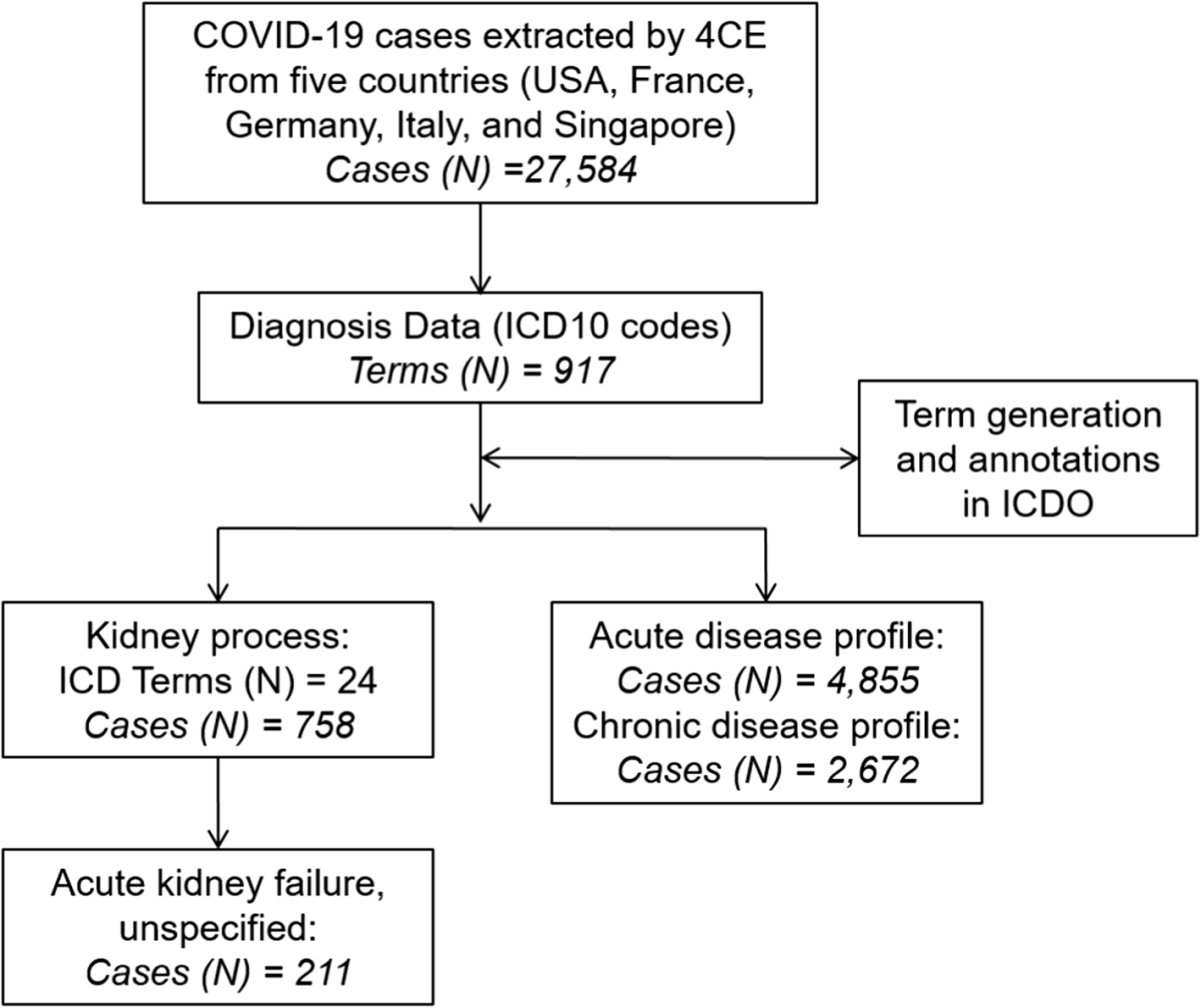
Workflow of 4CE COVID-19 clinical diagnosis data analysis

The diagnostic data provided by the Consortium for Clinical Characterization of COVID-19 by EHR (4CE) [9] was downloaded from their website (https://covidclinical.net/data/index.html). The workflow of the 4CE diagnosis data analysis is shown in Fig. 1. Basically, the 4CE diagnosis data, which included 27,584 COVID-19 cases from five countries (USA, France, Germany, Italy, and Singapore), were downloaded on 5 July 2020. A total of 915 ICD-10 and ICD-9 codes were used to classify these COVID-19 cases. In our study, we mapped these codes to ICDO. If the ICDO did not have the terms, we then applied the ICDO development methods as described above to add the corresponding terms to ICDO and add new annotations as well. The mapped ICDO terms were then used to support further analysis of the ICD codes. As use case studies, we focused on kidney disease processes and various acute versus chronic disease profiles (Fig. 1).

### ICDO query and analysis

Description Logic (DL) query was used to query the knowledge built in ICDO. The DL query function in the Protégé-OWL editor was used for the implementation.

## Results

### General disease definition of disease development strategy

First, we performed a survey on how the term “disease” is defined in different ontologies and dictionaries (Table 1). It is clear that the nature of the disease is defined differently. In four ontologies, including DOID, OGMS [15], MONDO, and EFO (Experimental Factor Ontology) [22, 23], the disease is all defined as a disposition. In the Semanticscience Integrated Ontology (SIO) [24], the disease is defined as an outward manifestation of one or more disorders. The disease has also been defined as a disorder by itself or a pattern of abnormality (Table 1).

**Table 1:** Survey of disease definitions

Source	Definition
DOID, OGMS, and MONDO	A disease is a disposition (1) to undergo pathological processes that (4) exists in an organism because of one or more disorders in that organism
EFO	A disease is a disposition that describes states of disease associated with a particular sample and/or organism
SIO	disease is the outward manifestation of one or more disorders
Exposure Ontology [34]	A disease is a pattern of abnormal functioning, or abnormal localization of normal functioning, and/or abnormal localization of constituents when compared to other members of that species
Dictionary (https://www.dictionary.com)	A disorder of structure or function in a human, animal, or plant, especially one that produces specific signs or symptoms or that affects a specific location and is not simply a direct result of physical injury

In OGMS, there are two disease-related terms, ‘disease course’ and ‘pathological bodily process’. The term ‘disease course’ is defined as “the totality of all processes through which a given disease instance is realized”. However, it is unclear what the “all processes” in the definition stands for. It is possible that some of the processes are not directly related to the term disease. The OGMS term ‘pathological bodily process’ is defined as “a bodily process that is clinically abnormal”. The diseases listed in ICDO have already happened, and are not an upcoming event. Given that the ICD is used primarily for post-disease recording and insurance filing purposes, we think that the disease in ICD is primarily meant to be a type of pathological bodily process; therefore, the disease in ICD can be better regarded as a “disease process” under OGMS ‘pathological bodily process’.

In ICDO, based on the nature of ICD and its applications, we focus on the representation of disease processes instead. Therefore, the term ‘disease process’ becomes our major term, which is defined in ICDO as follows:

Disease process =def. a pathological bodily process that occurs in a specific anatomic location, realizes a disease disposition, has abnormal bodily phenotype, and results in a pathological anatomical entity.

Therefore, all the specific diseases in ICDO are all defined as disease processes, which are different from other disease description frameworks. As a result, ICDO represents all disease names from ICD11, ICD10, GB/T14396 as disease processes, often abbreviated with the suffix “DP” in ICDO term labels.

In this study, ICDO is mainly used to standardize and interpret the codes from different ICD versions, leading to ICD code interoperability. ICDO aims to standardize clinical data from international multi-centers and also data generated under different ICD local and modified ICD versions in China. To support the general interoperability goal, we have included ICD10 and ICD11 terms in both English and Chinese languages in the ICDO.

### ICDO top-level structure and system design

**Fig. 2:**
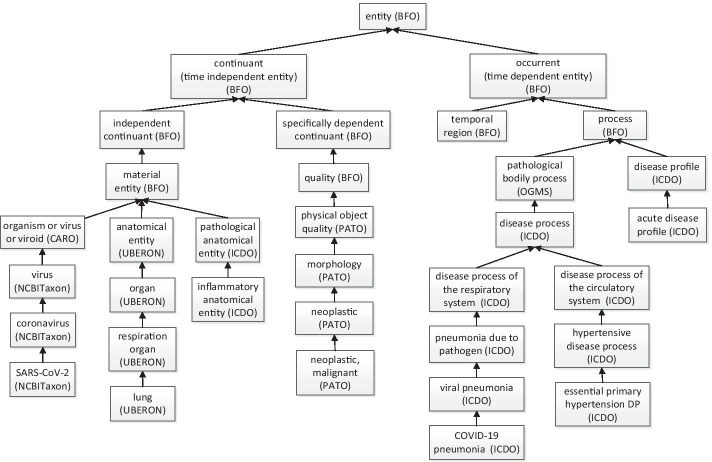
ICDO top-level hierarchical structure and selective terms. ICDO aligns with BFO ontology and reuses terms from many ontologies. ICDO also has many newly generated terms

While ICD10 and ICD11 have different classification principles, we have closely followed OBO to develop ICDO top-level hierarchy. Figure 2 provides the upper level hierarchical structure of the ICDO. First of all, ICDO is aligned with the Basic Formal Ontology (BFO) [8], an ISO-approved top-level ontology (https://www.iso.org/standard/74572.html). BFO includes two branches: ‘continuant’ and ‘occurrent’. Continuant is time-independent entities such as material entity, anatomical entity, quality, and role. Occurrent is time-dependent entities such as processes and time. As explained above, the ICDO *“disease process”* is defined as a process or occurrent (Fig. 2). In addition to BFO, ICDO also reuses terms from many existing ontologies such as the OGMS [15], UBERON [25], PATO (Phenotype And Trait Ontology, https://github.com/pato-ontology/pato/) (Fig. 2). The ICDO also generated many ICDO-specific terms, including those terms that are mapped to the ICD10, ICD11, and ICD9.

**Fig. 3:**
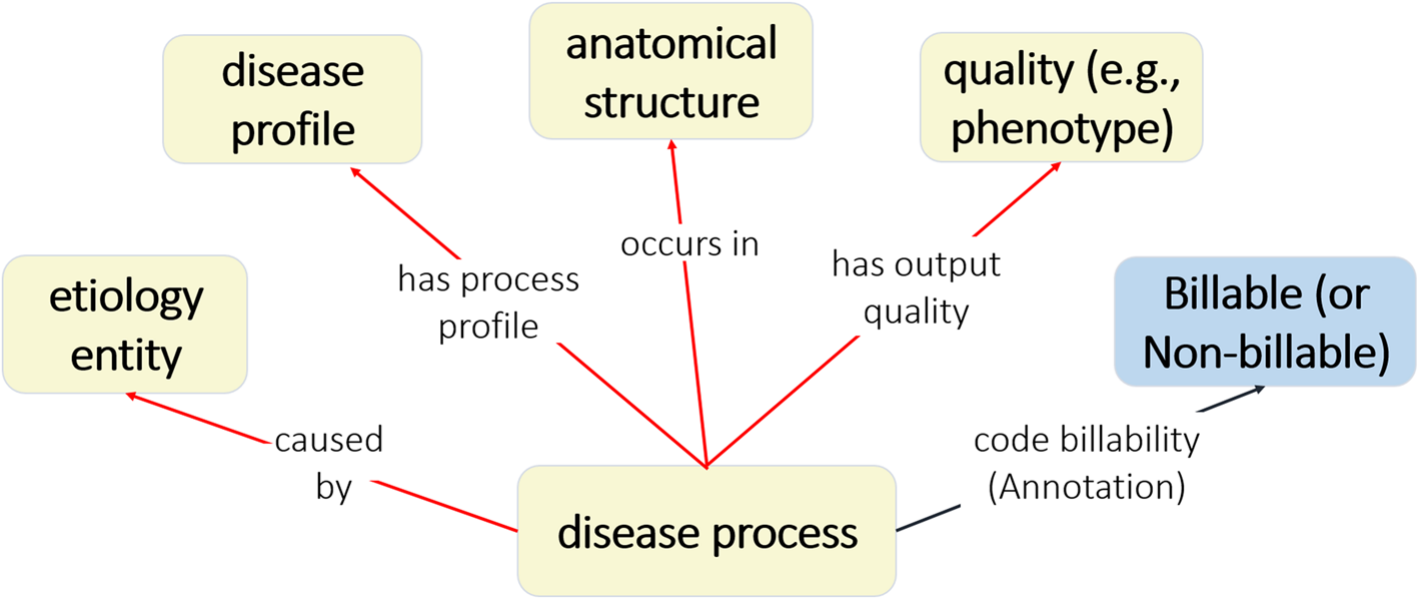
ICDO disease process pattern

In ICDO, a disease process was composed of four major elements: etiology entity, quality, anatomical structure, and pathological anatomical entity. The disease pattern of ICDO is shown in Fig. 3. Specifically, each disease process can be defined as having the following axioms:

- *‘occurs in’ some ‘anatomical location’*
- *‘has output quality’ some ‘quality (e.g., phenotype)’*
- *‘has process profile’ some ‘disease profile’*
- *‘caused by’ some ‘etiology entity’*

In addition, each disease process has an annotation ‘code billability’ which can have the value of “Billable” or “Non-billable.”

In ICD9/10, each term cannot have more than one parent term, and each term is placed in a branch restricted assigned with an alphabetic letter such as “A”. In reality, this rule meets many issues. For example, the ICD10 term ‘COVID-19 pneumonia’ has been assigned as the corresponding ICD code “U07.1”, which is under U07-U85 (Codes for special purposes). However, the viral disease may also be added under A00-B99 (Certain infectious and parasitic diseases), B25-B34 (Other viral diseases), or J00-J99 (Diseases of the respiratory system) (Table 2). This phenomenon demonstrates the difficulty and dilemma in terms of how to position a newly identified viral disease under a specific branch in ICD10.

**Table 2:** ICD10 classification of 4CE diagnosis data

Cases	ICD10 group terms	# of codes	# of billable codes	ICD10 group term labels
8825	J00-J99	51	33	Diseases of the respiratory system
5805	R00-R99	75	52	Symptoms, signs and abnormal clinical and laboratory findings, not elsewhere classified
3934	Z00-Z99	59	35	Factors influencing health status and contact with health services
1938	E00-E90	37	24	Endocrine, nutritional and metabolic diseases
1822	I00-I99	33	19	Diseases of the circulatory system
1056	B25-B34	9	4	Other viral diseases
1023	U07-U85	4	1	Codes for special purposes
701	N00-N99	15	12	Diseases of the genitourinary system
343	D50-D89	14	10	Diseases of the blood and blood-forming organs and certain disorders involving the immune mechanism
304	F01-F99	12	5	Mental, behavioral and neurodevelopmental disorders
206	G00-G99	10	3	Diseases of the nervous system
190	K00-K99	10	7	Diseases of the digestive system
169	O20-O29	12	12	Other maternal disorders predominantly related to pregnancy
115	A00-B99	13	7	Certain infectious and parasitic diseases
94	C00-D49	7	4	Neoplasms
91	M00-M99	5	3	Diseases of the musculoskeletal system and connective tissue
40	H00-H59	3	1	Diseases of the eye and adnexa
39	L80-L99	3	0	Other disorders of the skin and subcutaneous tissue
28	W00-W19	2	0	Slipping, tripping, stumbling and falls
26	Y95	1	1	Nosocomial condition
46	S00-T88	4	0	Injury, poisoning and certain other consequences of external causes

The results came from the summary of the 391 ICD10 terms associated with the 4CE dataset. Each of these terms is associated with 10 or more cases from the 4CE dataset

As a measure to solve the above issue, ICDO assigns randomized non-redundant code numbers. For example, we assigned ‘COVID-19 pneumonia’ a non-redundant ID (ICDO_0000148) that does not include a special letter representing a special assignment, and meanwhile, it can go under more than one parent group based on ontology assertion or inferencing.

Besides general disease classifications, ICD includes many special terms such as “classified elsewhere”, “other specified” and “unspecified”. ICDO has implemented special strategies to handle the mentioned special terms.

Those ICD terms containing “classified elsewhere” were treated as obsolete terms in ICDO. The definition of “classified elsewhere” is confusing because there is no obvious and proper disease category for “elsewhere”. We believe that the disease classification must be clear and consistent among various disease categories. To ensure the classification integrity, a disease term can be classified under multiple disease categories based on varying definitions and applications, but it should not be classified under an undefined category, “elsewhere”. To balance the mapping process among various ICD versions and proper handling of the undefined category, we added all the ‘disease classified elsewhere’ terms in ICDO but made them as obsolete terms in the ontology.

There are also many ICD terms labeled as “other specified”. Logically speaking, all ICD terms should be classified into specific classes, and there should not exist any ‘other’ class. This “other specified” term class can be considered as a logical error, and we can put all the terms under this class into their parent class. Usually, we generated an ICDO term “other specified” and put it under the obsolete to support mapping among existing ICD versions. To ensure the continuity of the various versions of the ICD in both conversion adaptation and data adaptation processes, this obsolete class term may still participate in the operation to ensure the accuracy of data mapping. However, the “other specified” may have its specific meaning, and sometimes we would like to keep them but provide its specific annotation.

Many ICD terms, such as “Unspecified kidney failure” (ICD10 code: N19), are labeled as “Unspecified”. Using the label “unspecified”, a term is aligned in parallel with the other specific terms under the same parent term, but this term has no specific feature that differentiates it from the other terms under the same parent term due to different reasons such as the lack of knowledge. In this case, we may keep this term since the term offers more details than their parent term per se. These terms, including the N19 term, are also often “Billable” terms.

### ICD- and ICDO-based representation and analysis of COVID-19 disease data

As of 5 July 2020, the data collected by 4CE included 27,584 COVID-19 cases (Fig. 1), which have four sets of data (daily counts, demographics, labs, and diagnoses) from five countries (USA, France, Germany, Italy, and Singapore) [9]. We only used the diagnoses data that includes ICD10 or 9 codes and their associated case reports. Most of these ICD10 codes aligned with the ICD10-CM version (a version used in the USA, where CM means Clinical Modification). A total of 917 ICD terms were identified with at least 1 case found in 4CE. Table 2 provides an ICD10-based analysis of most of these codes, which shows the label of the group, as well as the numbers of cases, codes, and billability information per group. A term is considered “billable” if it is used to diagnose a patient for reimbursement purposes because it is the most specific code available to describe the disease. There are 514 “Billable” codes in this study.

According to Table 2, the three groups that have the most cases are R00-R99 (symptoms, signs, and abnormal clinical and laboratory findings, not elsewhere classified), J00-J99 (respiratory diseases), and Z00-Z99 (factors influencing health status and contact with health services). These three groups cover 18,564 cases in total. Based on this information, we came to the conclusion that COVID-19 can attack various different parts of the body. The three most affected systems are the respiratory, endocrine, and circulatory systems, of which cover 12,585 cases.

A few problems with the ICD system were also identified. In addition to unclear term labels, such as “not elsewhere classified” or “other”, we have observed that it is difficult to locate the diseases in the ICD hierarchy because of its one-dimensional nature hierarchy that does not allow a term to have more than one parent. For instance, when we were trying to sort the terms based on location, we found that the majority of respiratory diseases fell under the group J00-J99. However, we also realized that terms such as “dependence on respirator” and “severe acute respiratory syndrome, unspecified”, which actually are respiratory conditions, can be found out in the group Z00-Z99 and in the U07-U85 group, respectively. A final major problem we found regarded the elasticity of the ICD system and how it can be used in different situations. For example, U07.1, the new ICD10 code for “COVID-19 pneumonia”, is located under “Codes for special purposes (U07-U85)”, and is associated with 796 cases in 4CE. This is an issue because within a few years “COVID-19 pneumonia” is not a special case code anymore. That means the code needs to be changed accordingly. However, it is very hard to change the code from U07.1 to a different code that does not start from “U”. This case indicates that the ICD system is not flexible, and a code naming strategy in ICDO appears more appropriate to accommodate essential changes.

**Fig. 4:**
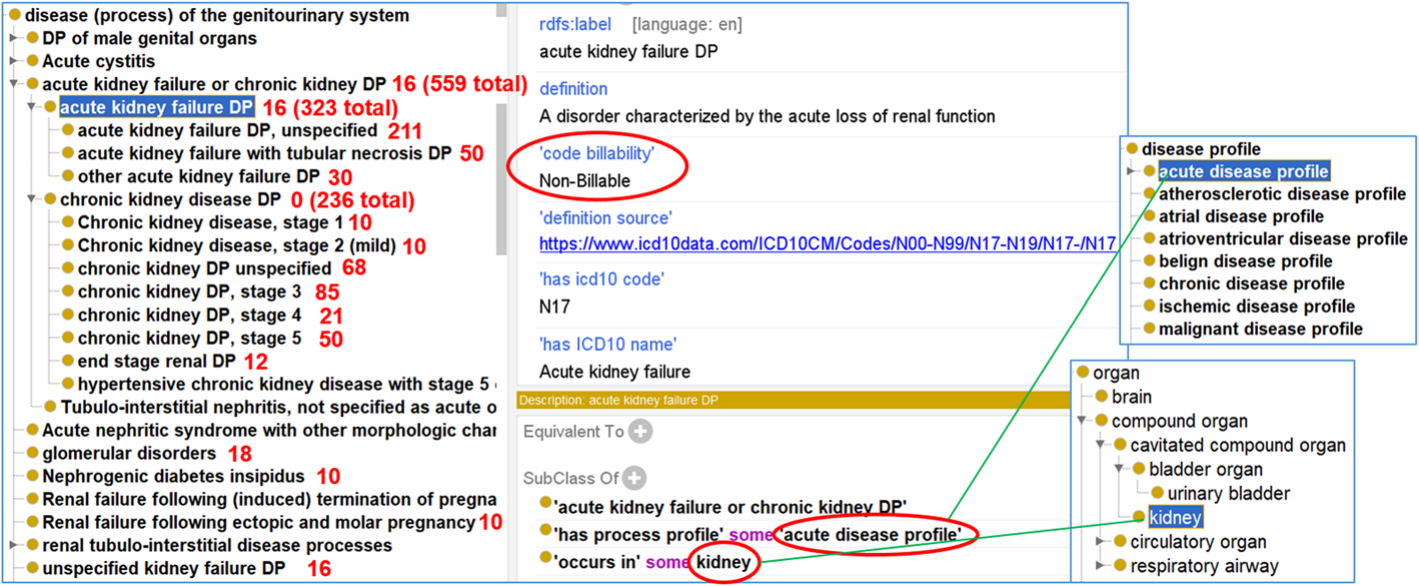
ICDO hierarchical class showing different types of kidney diseases and their associated case numbers. In this example, the term ‘acute kidney failure DP’ is represented using the design pattern, including disease profile, anatomical location, and billability

ICDO is designed to solve many issues in the ICD10 system. Figure 4 illustrates how ICDO represents the ICD term ‘acute kidney failure’, its annotations, and the hierarchy that contains this and other ICDO terms. By following the ICDO design pattern (Fig. 3), the ICD term ‘acute kidney failure’ is defined in ICDO as ‘*acute kidney failure DP*’, which is a disease process that *‘occurs in’ some ‘kidney’* and *‘has process profile’ some ‘acute disease process’*. It also has a ‘*code billability*’ information of “Non-billable” (Fig. 4).

In Fig. 4, we also found three more subclasses of ‘acute kidney failure DP’, including ‘acute kidney failure DP, unspecified’ (N17.9, ICDO_0000267), ‘acute kidney failure with tubular necrosis DP’ (N17.0, ICDO_0000265), and ‘other acute kidney failure DP’ (N17.8, ICDO_0000266), where “DP” is added to these terms to represent ‘disease process’. All three terms are billable terms. Here the ‘other acute kidney failure’ is not well defined. This term (N17.8) may be used to specify conditions or terms like an acute renal failure due to ischemia or ischemic nephropathy, or post-renal renal failure. To be more specific, it would be better to define these specific conditions, which will be considered by ICDO.

**Fig. 5:**
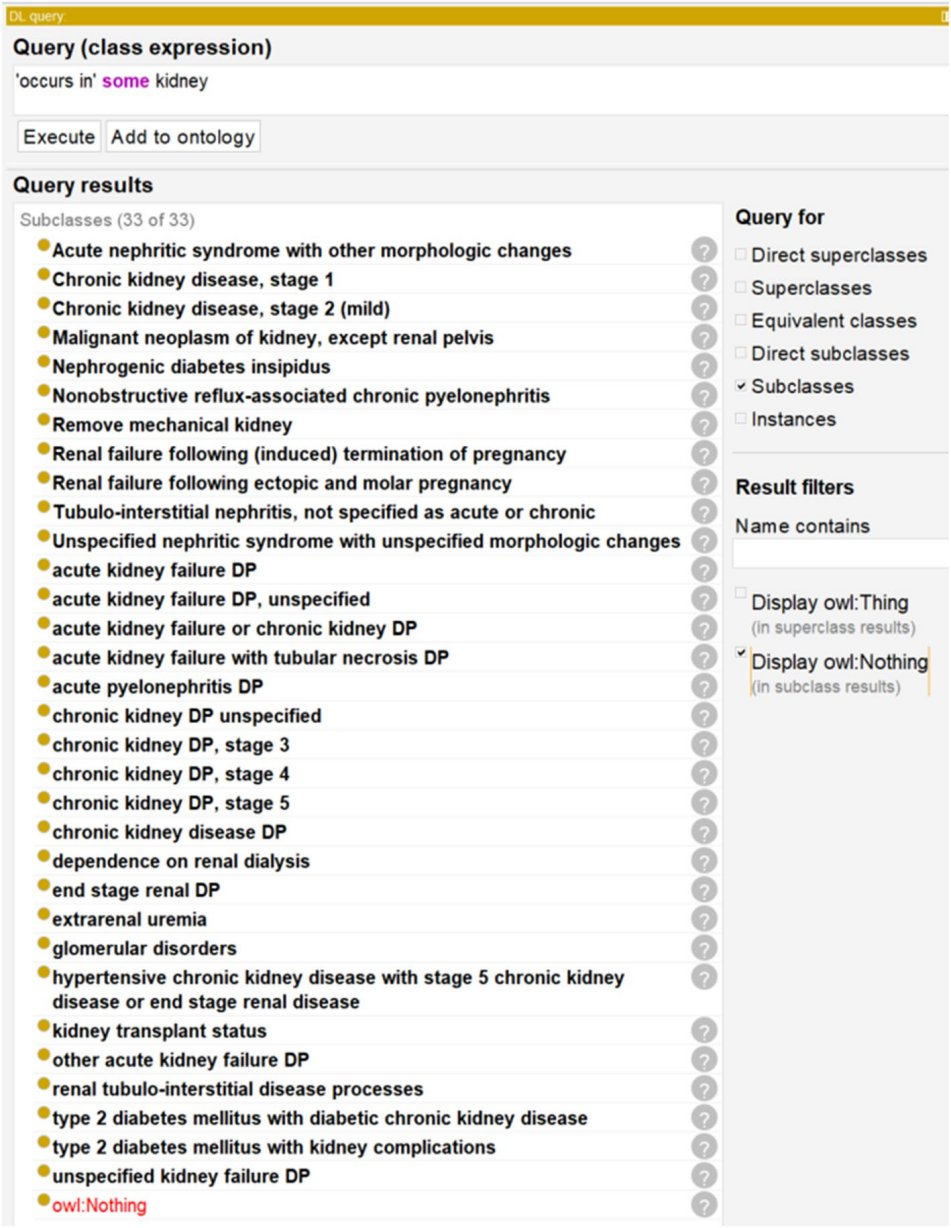
DL query of ICDO looking for all 4CE ICD codes that occur in the kidney. This query was performed using DL query in Protege-OWL editor 5.2

Figure 5 demonstrates how the ontology can be used to identify miscellaneous terms that occur at the kidneys using a Description Logic (DL) query. Basically, this DL query identified those diseases that meet this axiom requirement:

‘occurs in’ some kidney

The above axiom identified not only the kidney associated terms under the codes ranging from N00-N99, or “diseases of the genitourinary system”, but also codes that were found under other sections. These included codes E11.2 (type 2 diabetes mellitus with kidney complications), E11.22 (type 2 diabetes mellitus with diabetic chronic kidney disease), Z94.0 (kidney transplant status), Z99.2 (dependence on renal dialysis), and R39.2 (extrarenal uremia). This would not be possible with the ICD system since ICD10 does not include such axioms.

Our study further found 853 (3.1% of total) cases with 28 ICD codes that represent the disease processes in the kidney. Table 3 provides the detail of these 28 ICD codes and the cases associated with these codes. Most kidney-associated disease processes are under the groups of acute kidney failure and chronic kidney failure. The code with the highest number of cases is the “Acute kidney failure, unspecified” (N17.9), which is associated with 211 cases. In addition, there are two ICD codes (E11.2 and E11.22) that represent type 2 diabetes with kidney symptoms. Although these two ICD codes start with “E”, they do accompany kidney symptoms and so fits in with the criterion of occurring in the kidney. Therefore, the original letter-aligned ICD10 classification does not identify all disease processes in the kidney; however, our ICDO style of axiom definition can solve this issue.

**Table 3:** The ICD10 terms and their associated kidney associated case numbers in 4CE

ICD code	Case #	ICD label
N17	16	Acute kidney failure
N17.0	50	Acute kidney failure with tubular necrosis
N17-N19.9	16	Acute kidney failure and chronic kidney disease
N17.9	211	Acute kidney failure, unspecified
N18.9	68	Chronic kidney disease, unspecified
N18.1	10	Chronic kidney disease, stage 1
N18.2	10	Chronic kidney disease, stage 2 (mild)
N18.3	85	Chronic kidney disease, stage 3 (moderate)
N18.4	21	Chronic kidney disease, stage 4 (severe)
N18.5	50	Chronic kidney disease, stage 5
N17.8	30	other acute kidney failure
N19	16	Unspecified kidney failure
E11.2	30	type 2 diabetes mellitus with kidney complications
E11.22	13	Type 2 diabetes mellitus with diabetic chronic kidney disease
Z94.0	30	Kidney transplant status
Z99.2	11	Dependence on renal dialysis
R39.2	66	Extrarenal uremia
N18.6	12	End stage renal disease
N08.3	18	Glomerular disorders
O08.4	10	Renal failure following ectopic and molar pregnancy
O04.82	10	Renal failure following (induced) termination of pregnancy
N25.1	10	Nephrogenic diabetes insipidus
I12.0	10	Hypertensive chronic kidney disease with stage 5
N10	10	Acute pyelonephritis
N11.0	10	Nonobstructive reflux-associated chronic pyelonephritis
N12	10	Tubulo-interstitial nephritis, not specified as acute or chronic
N00.8	10	Acute nephritic syndrome with other morphologic changes
N05.9	10	Unspecified nephritic syndrome with unspecified morphologic changes

Similar to the above kidney disease searching, we can use the following axiom to identify which terms have the feature of acute or chronic disease profile:

*“has process profile” some ‘acute (or chronic) disease profile’*

Our analysis found 4,812 cases (17.84% of total) that have acute disease profile, and 2,622 (9.72% of total) cases that have chronic disease profile. As such, the conclusive perspective is that COVID-19 could cause more “acute” diseases than “chronic” ones.

## Discussion

In this manuscript, we presented our development of the ICDO ontology with the aim to standardize ICD disease records and support health record integration and analysis. We also proposed and tested a semantic analysis based on ICDO using the function of the reasoner, which interpreted terms at the semantic level by reasoner between entities by axioms. ICDO improves the mapping accuracy and supports exact and semantically preferred mapping. It also provides a useful application in terms of the standardization of heterogeneous data between different ICD versions. To demonstrate the usage of ICDO, our ICD-ICDO system was used to process and analyze the COVID-19 related diagnostic data available in the 4CE system.

One major theoretical contribution of the ICDO development is its establishment of the disease as a disease process. As detailed in this manuscript, diseases can be defined and classified in different ways. The OBO ontologies including the DOID and MONDO, define disease as a disposition. However, since ICD systems focus on the diagnosis of diseases that have historically occurred, it would be logical to treat the disease as a process instead of a disposition. Human Phenotype Ontology (HPO) [26] focuses on the classification of phenotypes instead of diseases. SNOMED CT is a systematically organized terminology of medical terms, which overlaps with ICD but differs in many ways [27]. The usage of SNOMED CT does not go with an open license. Instead, ICDO aims to closely map to ICD-10 and ICD-9 and later ICD-11 and it is developed as an open-source ontology. ICDO decomposes each disease terms into different components and formatted using the OWL, supporting semantic reasoning and inference.

We applied the ICDO to study the 4CE diagnosis data from thousands of COVID-19 patients in five countries. Our study found COVID-19 disease processes in different organs such as the kidney, showing that ICDO is capable of accurately sorting diseases based on anatomical location. Whereas the ICD system was not able to precisely summarize diseases that occurred in specific locations, the ICDO quickly solved this issue using the DL-Query. This feature can be applied for the several other dimensions of diseases allocated in ICDO, such as phenotype, etiology, and disease process profile.

Our study with the ICDO and 4CE dataset found that COVID-19 causes complications not only in the respiratory system, but other systems such as circulatory, digestive, and kidney systems as well. This may be likely because the cells in these systems all have angiotensin-converting enzyme 2 (ACE2), a receptor to which SARS-Cov-2 binds to invade cells [28]. For example, the ACE-2 receptor is expressed on the proximal tubules and glomeruli, which contribute to homeostasis and the filtration of the blood, respectively [29]. Damage to either of these can lead to kidney failure. However, different kidney phenotypes may not be all caused by SARS-CoV-2 infection since kidney disease can also be caused by other organ failures and other diseases such as diabetes. These results can help us to deepen our knowledge of the pandemic.

Indeed, our recent study observed two clinical phenotypes of acute kidney injury (AKI) in patients with COVID-19 and their risk factors and the association with mortality [30]. Using the clinical COVID-19 data from tertiary hospitals in China from 1 January to 23 March 2020, patients with AKI were classified to AKI-early and AKI-late according to the sequence of organ dysfunction (kidney as the first dysfunctional organ or not). These two clinical AKI phenotypes are likely attributed to two distinct mechanisms, viral sepsis or SARS-CoV-2 direct infection. Many factors such as viral infection, gender, age, host genetics, and patient disease history may contribute to the formation of these different AKI phenotypes. More systematic and integrative analyses are required for us to further define the risk factors of COVID-19-related kidney diseases and analyze the deep mechanisms under different phenotypes.

Note that our study focused on the ICDO ontology-based standardization and analysis of ICD-coded diagnosis data, and it missed the inclusion of many other data types (e.g., gender, age, and experimental data). The clinical ICD coding system assigns procedural and diagnostic codes specified in a medical classification system. The diagnostic and procedural codes are mainly used for reporting and reimbursement purposes of health care providers, which is the basic feature in a health care record. Basically, ICD codes are structured and standard data extracted from electronic health records (EHR), which are fundamental and critical for other research applications. The missing features in real word data do not affect the use of ICD codes. Meanwhile, the ICD coding results can be applied to integrate other features and data types, including clinical and experimental data, to support deep disease research.

Ontology is clearly a very good tool for solving the problem of semantic mapping between different ICD versions, which can even be established in different languages. ICDO will improve the usability and interoperability among various ICD systems. Since ICDO uses the Basic Formal Ontology (BFO) [8, 31] as the top level ontology, ICDO is interoperable with over 300 other BFO-aligned ontologies, such as the HPO and Coronavirus Infectious Disease Ontology (CIDO) [32, 33], thus facilitating integrative data representation and analysis. ICDO can also be used for data standardization and analysis of international multi-center clinical trials between different languages in different countries, data normalization processing before DGRs grouping, data normalization and in-hospital internal information systems, and data standardization for regional health information platforms. The disease design pattern in ICDO can provide effective contributions to medical data mining and retrospective researches.

For future study, the ICDO can be applied to represent other ICD codes and study other use cases, supporting more integrative and accurate organization of clinical diagnosis data and electronic health records. The overall coverage of ICDO is still relatively small. This paper provides a proof-of-concept demonstration of how the ICDO can be useful to study the COVID-19 data. After suggestions and comments are received, we will later move forward to increase the ICDO coverage. Another action is to collect and access the COVID-19 data in Chinese from China and apply this ICDO approach to do the analysis.

## Conclusions

We present our development of the ICD ontology (ICDO) for ontologization of ICD-10 and ICD-9 codes and the usage of the ICDO ontology to analyze the COVID-19 4CE diagnosis data. Compared to the ICD-9/10 and other existing disease ontologies, ICDO represent diseases as disease processes with many specific features including the etiological cause of the disease, anatomical location where the disease occurs, process profiles, and output patient qualities. Over 900 ICD terms have been represented in ICDO. The ICDO system was used to represent and analyze over 900 ICD codes used to represent the 4CE diagnosis data of over 27,000 COVID-19 patients from 5 countries. Our study found that COVID-19 caused various phenotypes and diseases in the lung and many other systems and organs such as the heart and kidneys. Many acute and chronic kidney phenotypes were identified. The kidney diseases were specifically analyzed. Our results showed that COVID-19 related kidney diseases could also result from other non-kidney diseases such as diabetes, which are not classified by default under the ICD category of kidney diseases. The ontological representation in ICDO supports efficient semantic reasoning and queries. By increasing the number of data sources and types, the ICDO coverage will be increased in future work. Therefore, ICDO offers many advanced features compared to the original ICD system and supports standardized diagnostic data integration and semantic reasoning on diseases such as COVID-19. The ICDO ontology will also be further developed with increased coverage or features in the near future.

## Data Availability

The current ICDO source code is openly available at GitHub: http://github.com/icdo/ICDO. The COVID-19 diagnosis data was downloaded from the 4CE website (https://covidclinical.net/data/index.html).

## Abbreviations

4CE: Consortium for Clinical Characterization of COVID-19 by EHR
AKI: Acute kidney injury
BFO: Basic Formal Ontology
CIDO: Coronavirus Infectious Disease Ontology
COVID-19: Coronavirus disease 2019
DL: Description Logic
DOID: Human Disease Ontology
DP: Disease process
DRG: Diagnosis-related group
EFO: Experimental Factor Ontology
EHR: Electronic health record
EMR: Electronic medical records
HIS: Health information systems
HPO: Human Phenotype Ontology
ICD: International Classification of Diseases
ICD10-CM: International Classification of Diseases 10-Clinical Modification
ICDO: International Classification of Diseases Ontology
LIS: Laboratory information system
MONDO: Monarch Disease Ontology
NCBO: National Center for Biomedical Ontology
OBO: Open Biological and Biomedical Ontology
OGMS: Ontology of General Medical Science
OWL: Web Ontology Language
PACS: Picture archiving and communication system
PATO: Phenotype And Trait Ontology
SIO: Semanticscience Integrated Ontology
UBERON: Uber-anatomy ontology
WHO: World Health Organization
XOD: EXtensible Ontology Development
